# The association between air pollution and the daily hospital visits for atrial fibrillation recorded by ECG: a case-crossover study

**DOI:** 10.1186/s40001-023-01170-y

**Published:** 2023-06-29

**Authors:** Jiming Han, Rui Zhang, Jingyi Guo, Qingfeng Zheng, Xin Chen, Shanmei Wu, Jianguo Tan, Yongguang Li

**Affiliations:** 1 Department of Cardiology, Shanghai Sixth People’s Hospital Affiliated to Shanghai Jiao Tong University School of Medicine, 600 Yishan Rd, Shanghai, 200233 People’s Republic of China; 2 Department of Orthopaedics, Shanghai Sixth People’s Hospital Affiliated to Shanghai Jiao Tong University School of Medicine, 600 Yishan Rd, Shanghai, People’s Republic of China; 3 Department of Clinical Research Center, Shanghai Sixth People’s Hospital Affiliated to Shanghai Jiao Tong University School of Medicine, 600 Yishan Rd, Shanghai, People’s Republic of China; 4 Shanghai Key Laboratory of Meteorology and Health, Shanghai Meteorological Service, 280 Caoxi North Rd, Shanghai, 200030 People’s Republic of China; 5 Shanghai Jiao Tong University School of Medicine, 227 Chungking South Rd, Shanghai, People’s Republic of China

**Keywords:** Air pollution, Atrial fibrillation, Gaseous air pollutant, Particulate matter

## Abstract

**Background:**

The relationship between air pollution and atrial fibrillation (AF) recorded by electrocardiograph (ECG) has not yet been illustrated which worsens AF precaution and treatment. This research evaluated the association between air pollution and daily hospital visits for AF with ECG records.

**Methods:**

The study enrolled 4933 male and 5392 female patients whose ECG reports indicated AF from 2015 to 2018 in our hospital. Such data were then matched with meteorological data, including air pollutant concentrations, collected by local weather stations. A case-crossover study was performed to assess the relationship between air pollutants and daily hospital visits for AF recorded by ECG and to investigate its lag effect.

**Results:**

Our analysis revealed statistically significant associations between AF occurrence and demographic data, including age and gender. This effect was stronger in female (*k* = 0.02635, *p* < 0.01) and in patients over 65 y (*k* = 0.04732, *p* < 0.01). We also observed a hysteretic effect that when exposed to higher nitrogen dioxide(NO_2_), counting AF cases recorded by ECG may elevate at lag 0 with a maximum odds ratio(OR) of 1.038 (95% CI 1.014–1.063), on the contrary, O_3_ reduced the risk of daily visits for AF and its maximum OR was at lag 2, and the OR value was 0.9869 (95% CI 0.9791–0.9948). Other air pollutants such as PM_2.5_, PM_10_, and SO_2_ showed no clear relationship with the recorded AF.

**Conclusion:**

The associations between air pollution and AF recorded with ECG were preliminarily discovered. Short-term exposure to NO_2_ was significantly associated with daily hospital visits for AF management.

**Supplementary Information:**

The online version contains supplementary material available at 10.1186/s40001-023-01170-y.

## Introduction

The associations between air pollutant concentration and its adverse effects on cardiovascular system, especially cardiac arrhythmias, have been illustrated by numerous reports [1–4]. However, only a few researchers have tried to decipher the relationship between air pollution and AF.

The meta-analysis, carried out by Shao et al. issued a statistically significant association between AF development and certain gaseous pollutant, such as nitrogen monoxide (NO), carbon monoxide (CO), sulfur dioxide (SO_2_) and ozone (O_3_), as well as particulate matter (PM) [5]. Through the investigation of the association between daily emergency ambulance calls (EC) for paroxysmal atrial fibrillation and air pollution, Vencloviene et al. pointed out a short-term hysteretic effect in people over 65 years old who were exposed to CO and PM_10_ and developed AF [6]. Meanwhile, another 14-year time-series study found the evidence that increased PM concentration was associated with AF prevalence after short-term exposure, and the effect was amplified in the case of female gender and the elderly [7].Besides, Monrad M. et al. found a long-term residential traffic-related air pollution exposure could be associated with higher risk of AF [8]. Increased ambient O_3_ was also considered one of the precipitant of paroxysmal AF [9]. Nevertheless, some researches pointed out that PMs were involved in the arrhythmia, while other researches verified that hospitalization for AF was not significantly associated with short-term exposure to elevated PM_2.5_, nor was there a correlation between air pollution and acute-onset AF [10–12]. Therefore, it is particularly important to investigate the relationship between pollutants and acute-onset AF. In this study, we investigated the correlation between pollutants and acute-onset AF recorded by ECG. Subgroup comparisons were also carried out to explore people who were more vulnerable to short-term air pollutants exposure.

## Methods

### Study population

AF, coding I48. × 01 in 10th edition of International Classification of Diseases (ICD-10), was recorded by ECG. Generally, the ECG changes of typical AF are characterized by irregularly irregular R–R intervals, absence of distinct repeating P waves and irregular atrial activation (ESC guidelines definition). Patients’ data were eligible if they were testified with ECG indicating AF from Jan 1st 2015 to Dec 31st 2018 in Shanghai Sixth People’s Hospital. This case-crossover study involving human participants was in accordance with the ethical standards of 1964 Helsinki Declaration and its later amendments or comparable ethical standards. The study was also approved by the ethics committee of Shanghai Sixth People’s Hospital. The study consisted of 4933 male and 5392 female patients. The study did not include minors (the youngest person with AF was 19 years old). Total of 10,863 AF cases recorded by ECG were obtained and 538 cases repeated by ECG in 48-h were excluded. Demographic data included name, gender, age, and we collected information of ECG (ST-T: ST-T changes; FVR: fast ventricular rate; SVR: slow ventricular rate; CB: conduction block; NSVT: nonpersistent supraventricular tachycardia; VPB: ventricular premature beat). All data were sorted in a date-based ascending order and were organized as gender and age group distribution. A single-centered database of AF with demographic data and data of ECG was then established.

### Environmental data

The daily averaged concentrations in Shanghai during the study period for fine particle mass and gaseous air pollutant, including PM_2.5_, PM_10_, O_3_, SO_2_, NO_2_ and CO, were obtained from Environmental Monitoring Center in Shanghai. To quantitatively describe short-term air quality conditions and trends in the study area, air quality index (AQI) was introduced, ever since AQI indicators were implanted in China in 2012. The referencing standards for AQI grading calculation are “Environmental Air Quality Standards” (GB3095-2012) and “AQI Technical Regulations (Trial)” (HJ633-2012). The AQI is calculated as follows:

AQI = max (IAQI1, IAQI2, IAQI3, …, IAQIn). In this formula, IAQIn refers to the air quality sub-index and n refers to each pollutant. The values of AQI in different intervals represent a specific air quality grade (Additional file 1: Table S1). When AQI is greater than 50, the pollutant with the highest IAQIn is defined as the primary pollutant; if the pollutant of the highest IAQIn is a tie between two or among more, they are then listed as primary pollutants. The monthly and quarterly mean air pollutant concentration was calculated based on daily mean concentration in Shanghai.

### Statistical analysis

Chi-squared tests and two-sample t-tests were applied to compare the demographic distribution of the two groups when appropriate. The Pearson correlation coefficient was used to analyze the association between AF and each air pollutant. A total of 10,325 AF in Shanghai Sixth People’s Hospital, from 2015 to 2018 was used to fit the model after adjusting for meteorological variables under the case-crossover analysis with the same year–month time stratification. Since each case serves as his or her own control, individual-level confounding factors that remain constant over a short period of time (e.g., age, race, gender, socioeconomic status) are controlled. The date of hospital visits served as the case day (lag 0). The association between lag 0–3 air pollutant concentrations and daily hospital visits for AF recorded by ECG was presented as an odds ratio (OR) with a 95% CI, and was conducted mathematically with a conditional logistic regression model using R (version 4.1.1; R Development Core Team, Vienna, Austria) with the season package. In order to make sure the relationship of NO_2_ and hospital visits for AF, conditional Logistic regression (R software "season" package) was used to analyze the influence of different lag pollutants on the number of daily AF, including PM_2.5_, PM_10_, O_3_, SO_2_, NO_2_ and CO and adjusted for weather factors (mean atmospheric pressure, mean temperature, mean relative humidity, mean wind speed). The parameters of the “lag” are defined in Additional file 2: Table S2. The statistical tests were two-sided, and the effects of *p* < 0.05 or *p* < 0.01 were considered statistically significant.

## Results

### The basic characteristics of study population and air pollutants

**Fig. 1:**
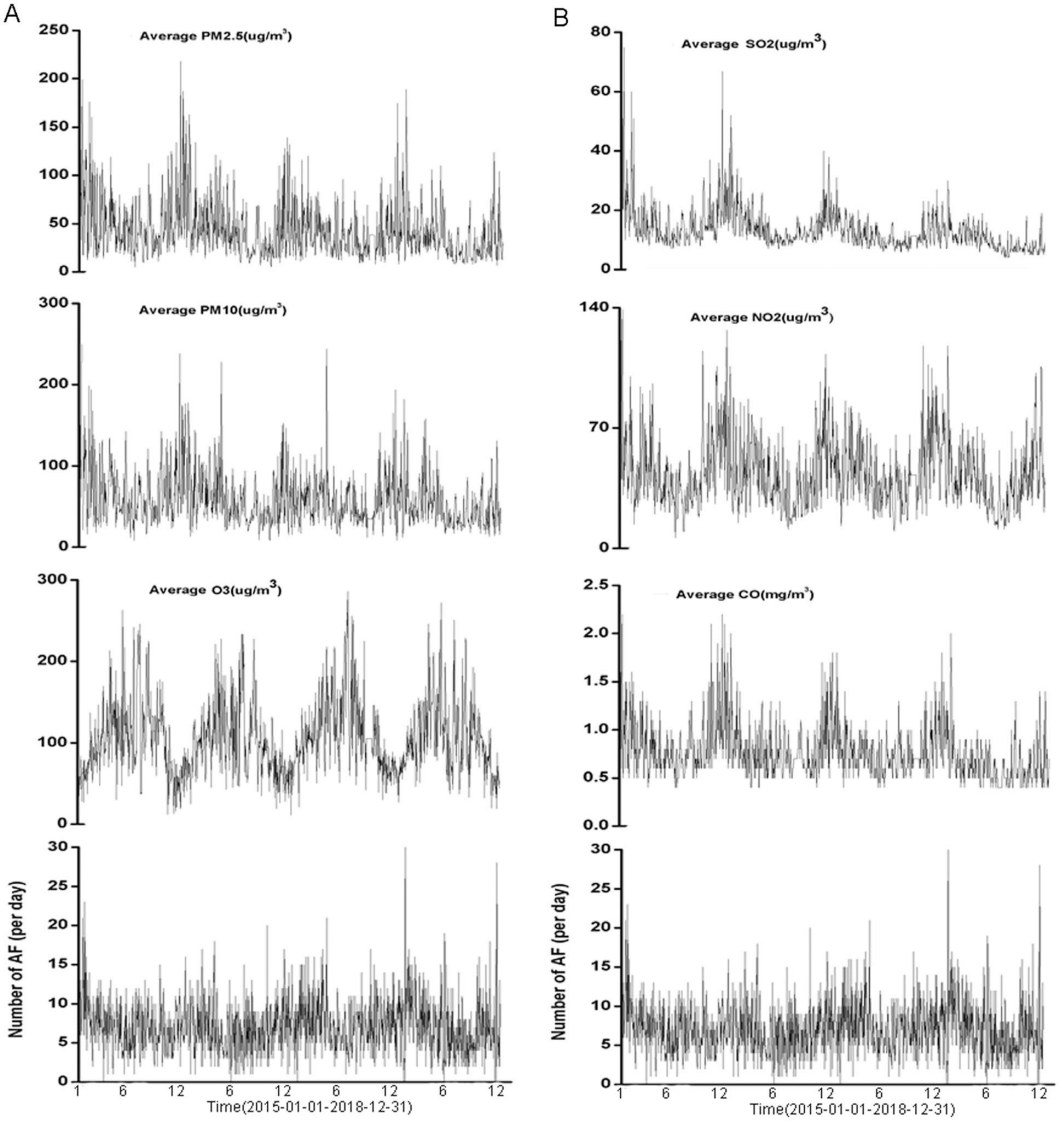
Daily data of AF recorded by ECG and the prevalence of AF with air pollution from 2015 to 2018. **A** Shows the PM_2.5_, PM_10_, O_3_ and the daily visits of AF; **B** shows the SO_2_, NO_2_, CO and the daily visits of AF

The AF recorded by ECG and the corresponding daily air pollutants are shown in Fig. 1. Air pollutants repeated regularly, but the concentration of air pollutants decreased year by year from 2015 to 2018. The concentrations of PM_2.5_, PM_10_, SO_2_, NO_2_ and CO were highest in winter (data not shown). The summary of basic demographic statistics of the study population and air pollution is shown in Table1. The mean age of patients was 76.49 ± 11.46 years old with 83.00% participants over 65. The mean age of patients of different genders was 74.41 ± 12.18 years old in males and 78.39 ± 10.40 years old in females, respectively. The occurrence of AF was different in terms of gender and age, and most of the AF occurrence was in patients over 65 years old (data not shown). Comparison of monthly and quarterly hospital visits for AF recorded by ECG in this study could be seen in Additional file 3: Fig. S1A, B. The monthly hospital visits for AF recorded by ECG reached its peak in January (2015 and 2016) and February (2018). However, the peak time in 2017 was seen in April. Besides, we did observe a rebound of the number of AF in spring in any year. The monthly average quality of NO_2_ ranged from 30 to 80 µg/m^3^ (91.7%). The monthly average quality of SO_2_ ranged from 5 to 20 µg/m^3^ (91.7%). The monthly average quality of PM_10_ ranged from 30 to 65 µg/m^3^ (95.8%). The monthly average quality of PM_2.5_ ranged from 25 to 80 µg/m^3^ (91.7%). The monthly average quality of CO ranged from 12 to 27 µg/m^3^ (93.8%). The monthly average quality of O_3_ ranged from 25 to 90 mg/m^3^ (91.7%). Most of the air pollutants reached their highest concentrations in the first and fourth quarters annually, negatively devastating air quality in the same period. On the other hand, O_3_ reached its highest concentration in the summer season, probably due to higher temperature.

**Table 1:** Summary of basic descriptive statistics of the study population and air pollution

Variables	Values
Total patients	10,325
Male	4933 (47.78%)
Male, years	74.41 ± 12.18
Female, years	78.39 ± 10.40
Age, years	76.49 ± 11.46
< 41, years	34.40 ± 5.11
41–65, years	58.78 ± 5.78
> 65, years	80.41 ± 7.35
ST-T	5056 (48.97%)
FVR	3263 (31.60%)
SVR	328 (3.18%)
CB	2152 (20.84%)
NSVT	39 (0.38%)
VPB	1271 (12.31%)
PM2.5 (µg/m^3^)	43.02 ± 28.74
PM10 (µg/m^3^)	63.46 ± 36.95
O3 (µg/m^3^)	105.16 ± 45.79
SO2 (µg/m^3^)	13.26 ± 6.86
NO2 (µg/m^3^)	43.73 ± 19.91
CO (mg/m^3^)	0.77 ± 0.27

ST-T, ST-T changes; FVR, fast ventricular rate; SVR, slow ventricular rate; CB, conduction block; NSVT, nonpersistent supraventricular tachycardia; VPB, ventricular premature beat

### Pearson correlation coefficient for the association of each parameter

The air pollution and meteorological measurements are shown in Additional file 4: Table S3. Daily NO_2_ was positively correlated with CO (Pearson correlation coefficient, *r* = 0.703) and PM_2.5_ (*r* = 0.680). NO_2_ was also positively correlated with SO_2_ (*r* = 0.607) and PM_10_ (*r* = 0.264). On the contrary, O_3_ was negatively correlated with NO_2_ (*r* = − 0.201) and the weather factors were also correlated with the air pollutant measurements.

### The correlation of the daily AF recorded by ECG and the air pollutants

**Fig. 2:**
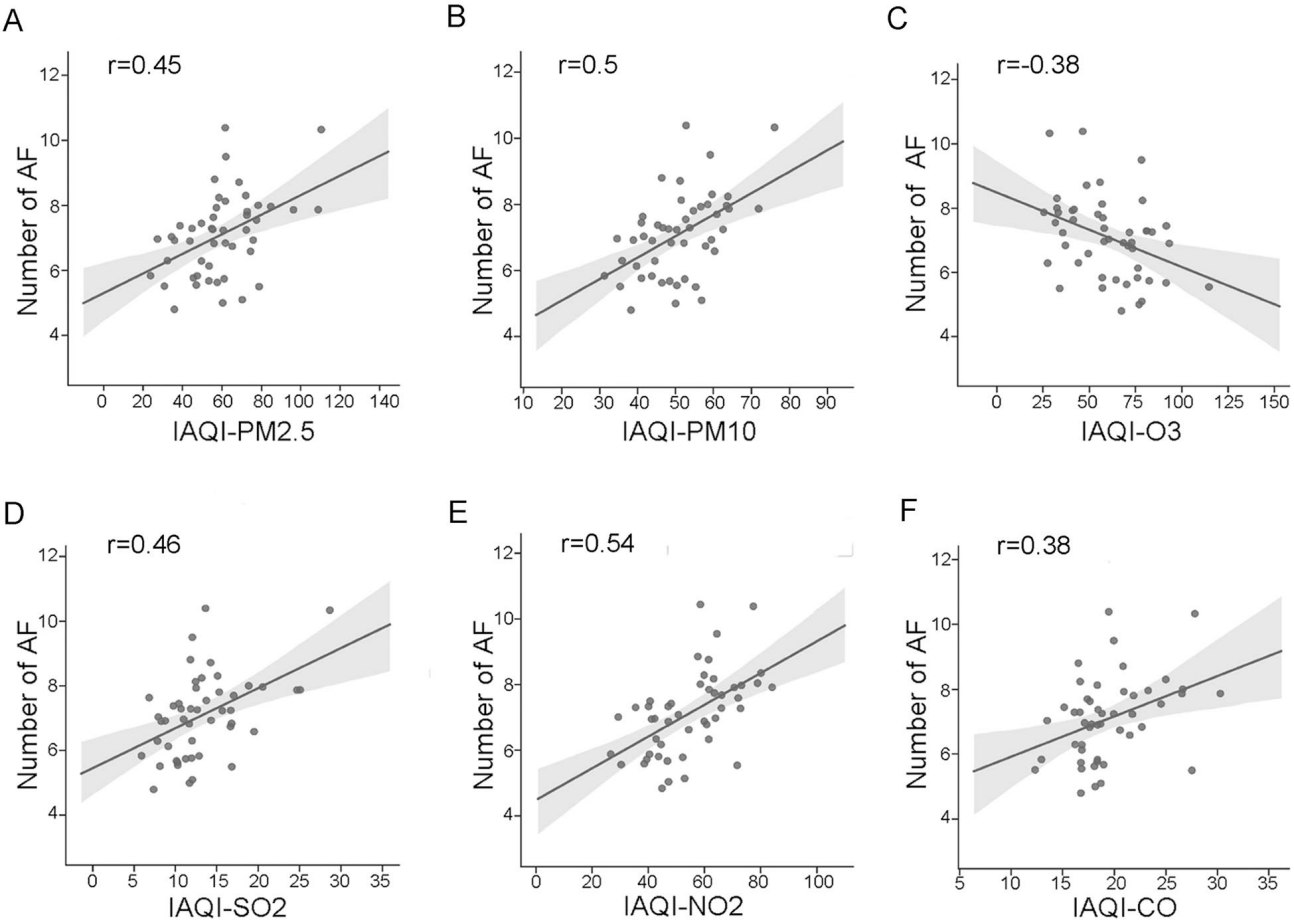
The relationship between IAQI and AF number recorded by ECG. **A**, **B** and **D**–**F** Show the positive correlation between AF recorded by ECG and each of the detected air pollutants (PM_2.5_ *r* = 0.45 *p* < 0.05, PM_10_ *r* = 0.50 *p* < 0.05, SO_2_ *r* = 0.46 *p* < 0.05, NO_2_ *r* = 0.54 *p* < 0.05, and CO *r* = 0.38 *p* < 0.01). **C** Shows a negative correlation of O_3_ with daily visits (*r* = − 0.38, *p* < 0.05)

**Fig. 3:**
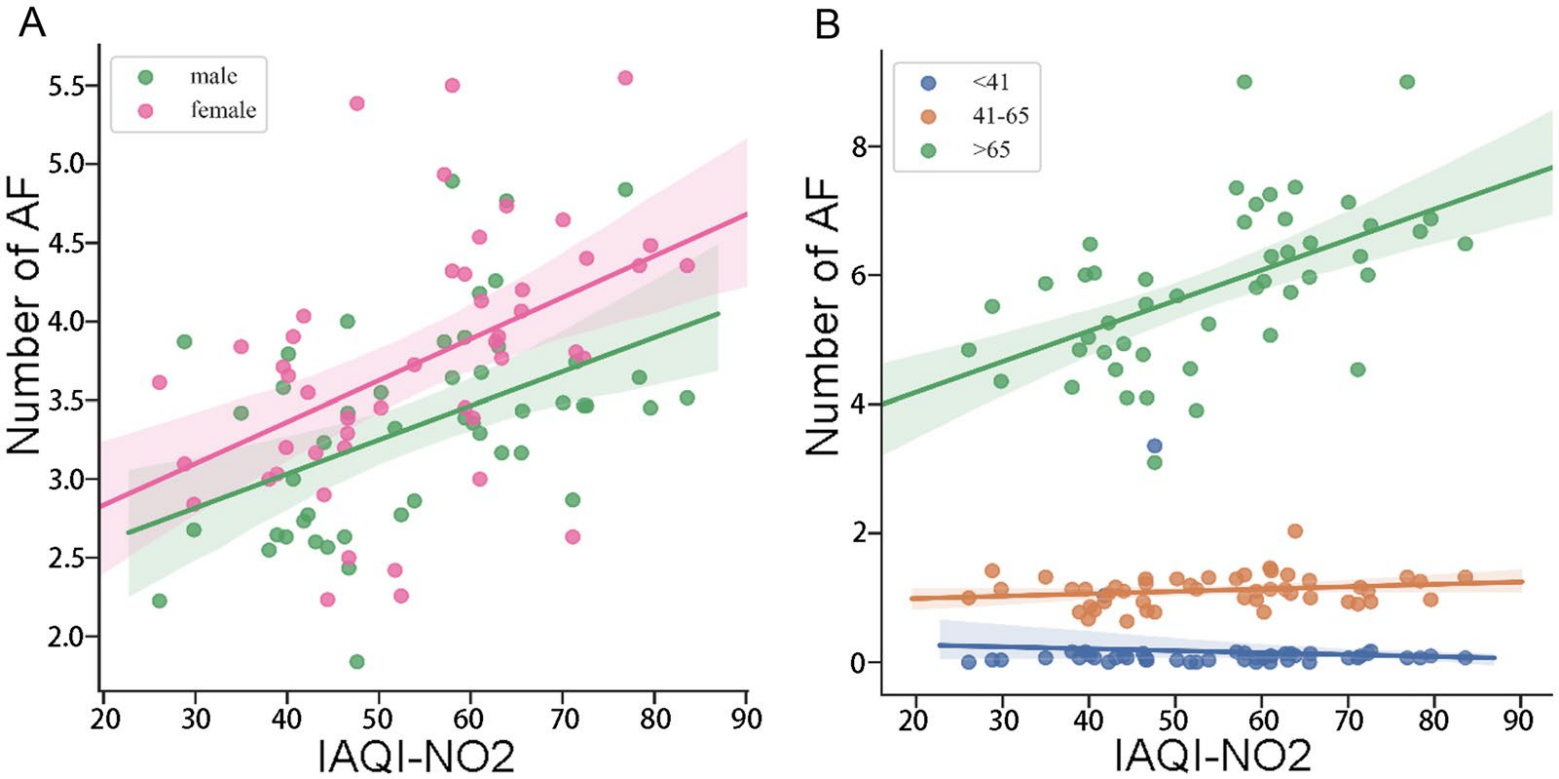
The AF patients with ECG in genders and age groups. **A** Shows that the slope of the regression curve in female patients (*k* = 0.02635, *p* < 0.01) was slightly sharper than in male patients (*k* = 0.02162, *p* < 0.01). **B** Shows that in age group analysis, the elderly patients over 65 years old were more vulnerable to air pollutants (*k* = 0.04732, *p* < 0.01)

For the monthly level, O_3_ showed a negative correlation with AF-onset daily visits (*r* = − 0.38, *p* < 0.05) and a positive correlation was seen between daily AF recorded by ECG and each of the rest of the detected air pollutants (Fig. 2a–f) (PM_2.5_ *r* = 0.45 *p* < 0.05, PM_10_ *r* = 0.50 *p* < 0.05, O_3_ *r* = − 0.38 *p* < 0.05; SO_2_ *r* = 0.46 *p* < 0.05, NO_2_ *r* = 0.54 *p* < 0.05, and CO *r* = 0.38 *p* < 0.01). Since NO_2_ showed the highest coefficiency on lag effects, we further tested if the influence was different in gender and age subgroups (Fig. 3a, b). While the slope of the regression curve in female patients (*k* = 0.02635, *p* < 0.01) was slightly sharper than that in male patients (*k* = 0.02162, *p* < 0.01), the hysteresis effect still remained in both genders. However, when it came to the age group fitting test, the hysteresis effect could only be seen in patients over 65 years old (*k* = 0.04732, *p* < 0.01). All results above were much more ambiguous when comparing weekly mean values of corresponding IAQI and AF. None of the absolute value of each Pearson correlation coefficient was above 0.20 (not shown).

### Air pollutants lag effects and AF recording

**Fig. 4:**
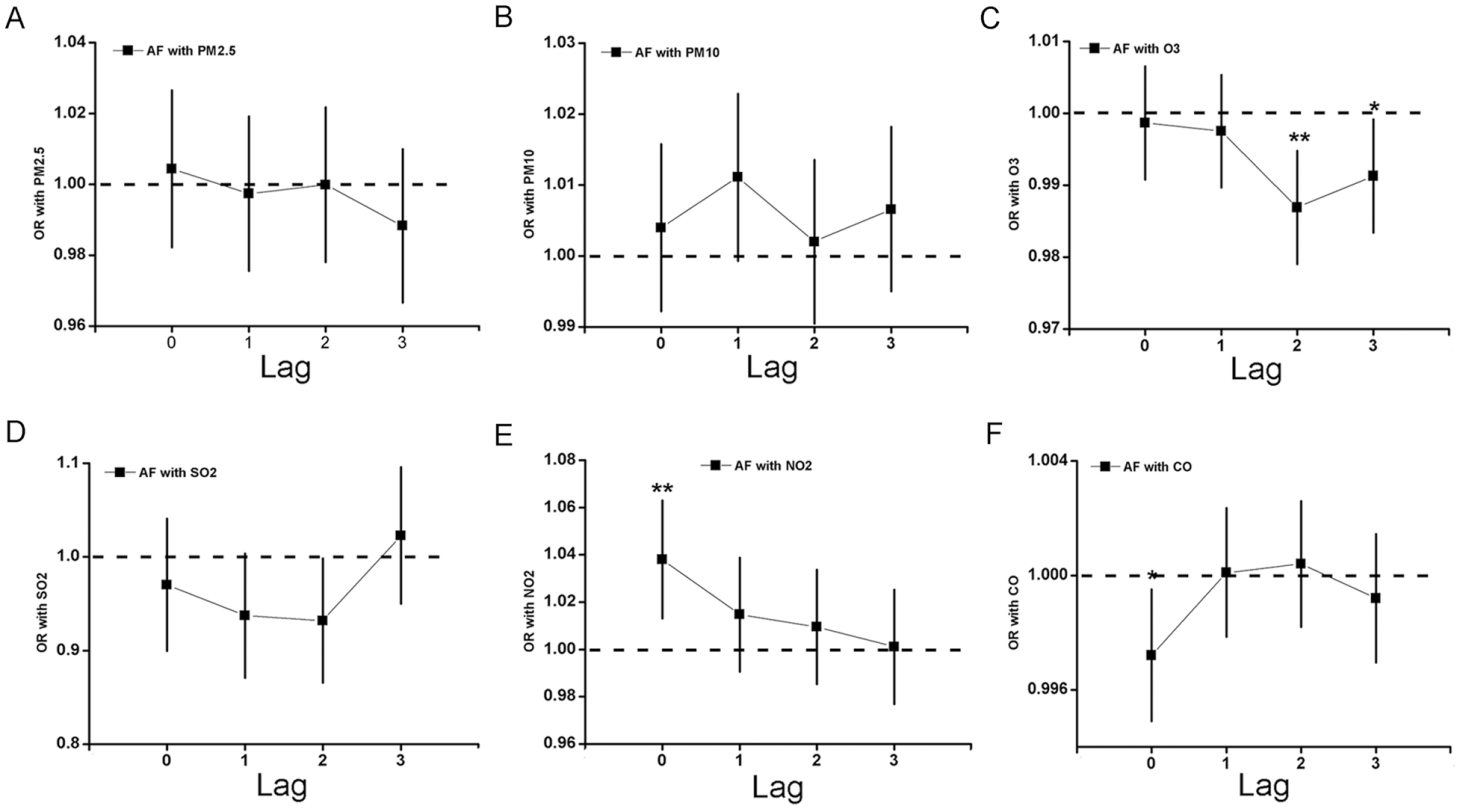
The case-crossover study was used to explore the correlation of AF recorded by ECG with air pollution. **A** Shows the AF with PM_2.5_; **B** shows the AF with PM_10_; **C** shows the AF with O_3_; **D** shows the AF with SO_2_; **E** shows the AF with NO_2_; **F** shows the AF with CO. *p < 0.05; **p < 0.01

To elucidate whether the air pollution could induce AF with lag effects, we analyzed the association between each air pollutant and the number of AF patients with ECG on day lag 0–3, respectively (Fig. 4). In order to make sure the relationship of NO_2_ and hospital visits for AF recorded by ECG, conditional logistic regression (R software "season" package) was used to analyze the influence of different lag pollutants on the number of daily AF recorded by ECG, including PM_2.5_, PM_10_, O_3_, SO_2_, NO_2_ and CO. And adjusted for weather factors (mean atmospheric pressure, mean temperature, mean relative humidity, mean wind speed) OR (95% CI). The PM_2.5_, PM_10_ and SO_2_ have no relationship with daily visits of AF recorded by ECG. But NO_2_ increases the risk of daily visits of AF recorded by ECG and the maximum OR at lag 0, and the OR value was 1.0381 (95% CI 1.0135–1.0634). The O_3_ reduces the risk of daily visits of AF recorded by ECG and the maximum RR at lag 2, and the OR value was 0.9869 (95% CI 0.9791–0.9948) and adjusted for weather factors, The CO reduces the risk of daily visits of AF recorded by ECG at lag 0, and the OR value was 0.9972 (95% CI 0.9949–0.9995) (Fig. 4a–f).

## Discussion

### Main findings

The most important finding of this study was that significant increase of daily hospital visits for AF patients with positive ECG was associated with increments in NO_2_ concentration at lag 0 in Shanghai. Also, this effect was stronger within female patients and patients over 65.

### Air pollutants were associated with AF recorded by ECG

Nowadays, air quality is one of the most popular issues to human life [13]. It has been reported that air pollution, low temperature, air humidity and air pressure changes could threaten certain population who are more vulnerable to cardiovascular diseases [1–4, 6, 14–19]. During this research, we proved that ambient environmental changes resulted in gender-related arrhythmia. We found that people over 65 years old had a higher daily hospital visits for AF recorded by ECG, which was consistent with previous studies [11]. In this research, we found that NO_2_ enhanced daily hospital visits for AF through the correlation between ECG records and meteorological pollution data. Though PM_10_ showed similar hysteresis effect, as it increased the risk of daily hospital visits of AF recorded by ECG, its devastating function was much weaker than NO_2_ and might need further detection [6]. Interestingly, the hysteresis effect of PM_2.5_ was unexpectedly weaker than that of PM_10_. O_3_ and CO needs more evidence to show their capabilities in inducing or inhibiting air pollution-related arrhythmia, because our results were opposite to the previous findings by Rich et al. [8], who demonstrated a positive relationship between O_3_ and CO concentrations and AF.

NO_2_ has a variety of both natural (volcano eruption, forest fire, etc.) and anthropogenic sources, derived from fossil fuel combustion (mainly originated from traffic and industrial consumption) [20]. Various studies have indirectly suggested short-term NO_2_ exposure would lead to cardiovascular mortalities, myocardial infarction, heart failure, and arrhythmias. Hoek et al. showed a significant correlation between air pollution and the daily mortality caused by arrhythmias in Netherlands [21–25]. Strong effect of NO_2_ inducing arrhythmias, including AF and increasing heart failure, has been described in Western Europe [26]. In Brazil, Santos et al. carried out a time-series study and reported the significant influence of concurrent-day NO_2_ concentration on the number of arrhythmia emergency room visits [27]. Zhao et al. also concluded that NO_2_ had larger and more robust effects on outpatient visits for arrhythmia than other pollutants [19]. A previous study led by Monrad M. et al. claimed that though long-term residential traffic-related air pollution could be associated with higher risk of AF, no clear regarding effect modification was seen between exposure to nitrogen oxides(NO_x_) and AF in the aspects of gender, hypertension or other risk factors [7]. However, in our study, gender and age were clear modifications for short-term NO_2_ exposure when exploring its hysteresis effect. Possible explanation for this difference could lie in later univariate analysis against population cognition towards typical arrhythmias and personal hospital visits behaviors. Hence, the mechanisms through which air pollutants cause AF need further research.

### The mechanism of air pollutants associated with arrhythmia

To our knowledge, the biological patterns of how air pollutants induce arrhythmia were still not fully understood. Most researchers believed that autonomic nerve system, inflammatory reaction, and direct damage of the gaseous factors might play an important role in the occurrence of AF mutually. Some hypothesized that exposure to PMs firstly caused acute imbalance of autonomic modulation, characterized by increased sympathetic and decreased parasympathetic nerve function, which led to accelerated conduction of myocardial cells. Certain bypass of susceptible population was then activated and consequently AF occurred. In other studies, controversial function changes of the autonomic nervous system were reported when exposing to air pollutants, suggesting that people with different susceptibility background react to air pollutant exposure variously. For instance, exposure to PMs is more likely to induce AF in people with cardiorespiratory disease [28–30]. Other pathways through which air pollutants lead to AF may include the direct adverse effect against cardiac electrophysiological pathways as well as decreased oxygen-carrying capacity of the blood [31, 32]. The latter may result in the constriction of vessels, especially coronary arteries, and ascend blood pressure at the same time or sequentially. Afterwards, damaged vessel endothelium malfunctions and oxidative stress injury occur, leading to arrhythmia [33]. Also, we observed a hysteretic effect on the AF occurrence after exposure to higher NO_2_. Thus, we believe that a potential and chronic mechanism exists if acute exposure to NO_2_ is confirmed to be associated with later AF by further research. Seaton et al. speculated that air pollutants, through activating a coagulation mechanism and elevating circulating inflammatory mediator emission, might result in a secondary pulmonary inflammation and a delayed but more severe cytokine storm [34]. The hysteretic consequence of this pathway was probably arrhythmia.

### Study limitation

This study does have several limitations. Since our hospital is the only center to perform this study in Shanghai, the conclusion of this study could represent the prevalence pattern of the eastern coastal area of China still needs more verification. Although case-crossover study was used, we excluded patients with AF who follow-up regularly, so we may have missed some patients of acute-onset AF who refused ECG.

## Conclusions

In conclusion, we observed a direct association between air pollutants, especially NO_2_ and AF patients with ECG occurrence in our hospital. Though greater adverse hysteresis effects were seen in the elderly and female patients, how these air pollutants induced the occurrence still remained unknown. Further researches may focus on the mechanisms of the interactions between air pollutants and autonomic neuron malfunction, coronary arterial inflammation and direct impact on the pacemaker cells. Overall, our findings could help establish an air quality forecast-based AF precaution system in East Asia.

### Supplementary Information

**Additional file 1: Table S1**. AQI grading and comparison of its different classifications in USA and PRC.

**Additional file 2: Table S2**. Parameters of the “lag” in this study.

**Additional file 3: Fig. S1**. Monthly and quarterly prevalence of AF recorded by ECG with air pollution from 2015 to 2018. We did observe a rebound of AF recorded by ECG in spring every year. And monthly incidence of AF recorded by ECG reached its peak in Januaryand February. However, the peak time in 2017 was seen in April. Besides, the monthly and quarterly concentration changes of PM_2.5_, PM_10_, O_3_, SO_2_, NO_2_ and CO from 2015 to 2018 were shown too.

**Additional file 4: Table S3**. The relationship of air pollution and meteorological measurements.

## Data Availability

The data set supporting the conclusions of this article is included within the article (and its additional file).

## References

[1] Mordukhovich I, Kloog I, Coull B (2016). Association between particulate air pollution and QT interval duration in an elderly cohort. Epidemiology.

[2] Kim IS, Sohn J, Lee SJ (2017). Association of air pollution with increased incidence of ventricular tachyarrhythmias recorded by implantable cardioverter defibrillators: vulnerable patients to air pollution. Int J Cardiol.

[3] Knezović M, Pintarić S, Mornar Jelavić M (2017). Correlation between concentration of air pollutants and occurrence of cardiac arrhythmias in a region with humid continental climate. Acta Clin Croa.

[4] Su C, Breitner S, Schneider A (2016). Short-term effects of fine particulate air pollution on cardiovascular hospital emergency room visits: a time-series study in Beijing, China. Int Arch Occup Environ Health.

[5] Shao Q, Liu T, Korantzopoulos P (2016). Association between air pollution and development of atrial fibrillation: a meta-analysis of observational studies. Heart Lung.

[6] Vencloviene J, Babarskiene RM, Dobozinskas P (2017). The short-term associations of weather and air pollution with emergency ambulance calls for paroxysmal atrial fibrillation. Environ Sci Pollut Res Int.

[7] Solimini AG, Renzi M (2017). Association between air pollution and emergency room visits for atrial fibrillation. Int J Environ Res Public Health.

[8] Monrad M, Sajadieh A, Christensen JS (2017). Long-term exposure to traffic-related air pollution and risk of incident atrial fibrillation: a cohort study. Environ Health Perspect.

[9] Rich DQ, Mittleman MA, Link MS (2006). Increased risk of paroxysmal atrial fibrillation episodes associated with acute increases in ambient air pollution. Environ Health Perspect.

[10] Bunch TJ, Horne BD, Asirvatham SJ (2011). Atrial fibrillation hospitalization is not increased with short-term elevations in exposure to fine particulate air pollution. Pacing Clin Electrophysiol.

[11] Liao D, Shaffer ML, He F (2011). Fine particulate air pollution is associated with higher vulnerability to atrial fibrillation—the APACR study. J Toxicol Environ Health A.

[12] Cervellin G, Comelli I, Lippi G (2013). Lack of correlation between air pollution and acute-onset atrial fibrillation. Can J Cardiol.

[13] Zhao A, Chen R, Kuang X (2014). Ambient air pollution and daily outpatient visits for cardiac arrhythmia in Shanghai, China. J Epidemiol.

[14] Culic V (2017). The association of air temperature with cardiac arrhythmias. Int J Biometeorol.

[15] Kim J, Kim H (2017). The association of ambient temperature with incidence of cardiac arrhythmias in a short timescale. Int J Biometeorol.

[16] Nguyen JL, Link MS, Luttmann-Gibson H (2015). Drier air, lower temperatures, and triggering of paroxysmal atrial fibrillation. Epidemiology.

[17] Kim J, Kim H (2017). Influence of ambient temperature and diurnal temperature range on incidence of cardiac arrhythmias. Int J Biometeorol.

[18] Hensel M, Stuhr M, Geppert D (2017). Relationship between ambient temperature and frequency and severity of cardiovascular emergencies: a prospective observational study based on out-of-hospital care data. Int J Cardiol.

[19] Zhao Q, Coelho M, Li S (2019). Temperature variability and hospitalization for cardiac arrhythmia in Brazil: a nationwide case-crossover study during 2000–2015. Environ Pollut.

[20] Hesterberg TW, Bunn WB, McClellan RO (2009). Critical review of the human data on short-term nitrogen dioxide (NO_2_) exposures: evidence for NO2 no-effect levels. Crit Rev Toxicol.

[21] Luo K, Li R, Li W (2016). Acute effects of nitrogen dioxide on cardiovascular mortality in Beijing: an exploration of spatial heterogeneity and the district-specific predictors. Sci Rep.

[22] Roswall N, Raaschou-Nielsen O, Ketzel M (2017). Long-term residential road traffic noise and NO_2_ exposure in relation to risk of incident myocardial infarction—a Danish cohort study. Environ Res.

[23] Sorensen M, Wendelboe Nielsen O, Sajadieh A (2017). Long-term exposure to road traffic noise and nitrogen dioxide and risk of heart failure: a cohort study. Environ Health Perspect.

[24] Santurtun A, Sanchez-Lorenzo A, Villar A (2017). The influence of nitrogen dioxide on arrhythmias in Spain and its relationship with atmospheric circulation. Cardiovasc Toxicol.

[25] Hoek G, Brunekreef B, Fischer P (2001). The association between air pollution and heart failure, arrhythmia, embolism, thrombosis, and other cardiovascular causes of death in a time series study. Epidemiology.

[26] Milojevic A, Wilkinson P, Armstrong B (2014). Short-term effects of air pollution on a range of cardiovascular events in England and Wales: case-crossover analysis of the MINAP database, hospital admissions and mortality. Heart.

[27] Santos UP, Terra-Filho M, Lin CA (2008). Cardiac arrhythmia emergency room visits and environmental air pollution in Sao Paulo, Brazil. Epidemiol Community Health.

[28] Gong H, Linn WS, Clark KW (2008). Exposures of healthy and asthmatic volunteers to concentrated ambient ultrafine particles in Los Angeles. Inhal Toxicol.

[29] Graff DW, Cascio WE, Rappold A (2009). Exposure to concentrated coarse air pollution particles causes mild cardiopulmonary effects in healthy young adults. Environ Health Perspect.

[30] Tunnicliffe WS, Hilton MF, Harrison RM, Ayres JG (2001). The effect of sulphur dioxide exposure on indices of heart rate variability in normal and asthmatic adults. Eur Respir J.

[31] Donaldson K, Stone V, Seaton A (2001). Ambient particle inhalation and the cardiovascular system: potential mechanisms. Environ Health Perspect.

[32] Mills NL, Amin N, Robinson SD (2006). Do inhaled carbon nanoparticles translocate directly into the circulation in humans?. Am J Respir Crit Care Med.

[33] Shahrbaf MA, Akbarzadeh MA, Tabary M (2021). Air pollution and cardiac arrhythmias: a comprehensive review. Curr Probl Cardiol.

[34] Seaton A, MacNee W, Donaldon K (1995). Particulate air pollution and acute health effects. Lancet.

