# Correction: The association between air pollution and the daily hospital visits for atrial fibrillation recorded by ECG: a case-crossover study

**DOI:** 10.1186/s40001-023-01266-5

**Published:** 2023-08-12

**Authors:** Jiming Han, Rui Zhang, Jingyi Guo, Qingfeng Zheng, Xin Chen, Shanmei Wu, Jianguo Tan, Yongguang Li

**Affiliations:** 1 Department of Cardiology, Shanghai Sixth People’s Hospital Affiliated to Shanghai Jiao Tong University School of Medicine, 600 Yishan Rd, Shanghai, 200233 People’s Republic of China; 2 Department of Orthopaedics, Shanghai Sixth People’s Hospital Affiliated to Shanghai Jiao Tong University School of Medicine, 600 Yishan Rd, Shanghai, People’s Republic of China; 3 Department of Clinical Research Center, Shanghai Sixth People’s Hospital Affiliated to Shanghai Jiao Tong University School of Medicine, 600 Yishan Rd, Shanghai, People’s Republic of China; 4 Shanghai Key Laboratory of Meteorology and Health, Shanghai Meteorological Service, 280 Caoxi North Rd, Shanghai, 200030 People’s Republic of China; 5 Shanghai Jiao Tong University School of Medicine, 227 Chungking South Rd, Shanghai, People’s Republic of China

**Correction: European Journal of Medical Research (2023) 28:201** **https://doi.org/10.1186/s40001-023-01170-y**

The article title in the original publication [1] was incorrectly given as “The associations between air pollution and the daily hospital visits for atrial fibrillation recorded by ECG: a cross-sectional study" but should have been “The associations between air pollution and the daily hospital visits for atrial fibrillation recorded by ECG: a case-crossover study". The original article has been corrected.
