# Supplementary material for: The association between air pollution and the daily hospital visits for atrial fibrillation recorded by ECG: a case-crossover study
Source: Eur J Med Res. 2023 Jun 29;28:201. doi: 10.1186/s40001-023-01170-y (PMC10308751; doi:10.1186/s40001-023-01170-y)
Supplement: Supplementary file 1 — Additional file 1: Table S1. AQI grading and comparison of its different classifications in USA and PRC. [file 40001_2023_1170_MOESM1_ESM.doc]

**Supplementary table1.** AQI grading and comparison of its different classifications in USA and PRC

| **AQI** | **Grade** | **Classification of certain AQI** | |
| --- | --- | --- | --- |
|  | **PRC version** | **USA version** | **PRC version** |
| 0~50 | I | Good | Excellent |
| 51~100 | II | Moderate | Good |
| 101~150 | III | Unhealthy for certain groups | Mild contamination |
| 151~200 | IV | Unhealthy | Middle contamination |
| 201~300 | V | Very unhealthy | Serious contamination |
| 301~500 | VI | Hazardous | Severe contamination |
| 501+ |  | Beyond index |  |

USA: United States of America. PRC: People’s Republic of China.
