# Supplementary material for: The association between air pollution and the daily hospital visits for atrial fibrillation recorded by ECG: a case-crossover study
Source: Eur J Med Res. 2023 Jun 29;28:201. doi: 10.1186/s40001-023-01170-y (PMC10308751; doi:10.1186/s40001-023-01170-y)
Supplement: Supplementary file 2 — Additional file 2: Table S2. Parameters of the “lag” in this study. [file 40001_2023_1170_MOESM2_ESM.doc]

**Supplementary table2.** Parameters of the “lag” in this study.

| **Lag n** | **Source of air pollutant concentration** | **Source of AF number** |
| --- | --- | --- |
| lag 0 | Day 0 | Day 0 |
| lag 1 | Day 0 | Day 1 |
| lag 2 | Day 0 | Day 2 |
| lag 3 | Day 0 | Day 3 |

The corresponding source of air pollutant was from Day 0. Source of AF number of the corresponding lag day could also be checked through this table.
