# Supplementary figures and images for: The association between air pollution and the daily hospital visits for atrial fibrillation recorded by ECG: a case-crossover study
Source: Eur J Med Res. 2023 Jun 29;28:201. doi: 10.1186/s40001-023-01170-y (PMC10308751; doi:10.1186/s40001-023-01170-y)

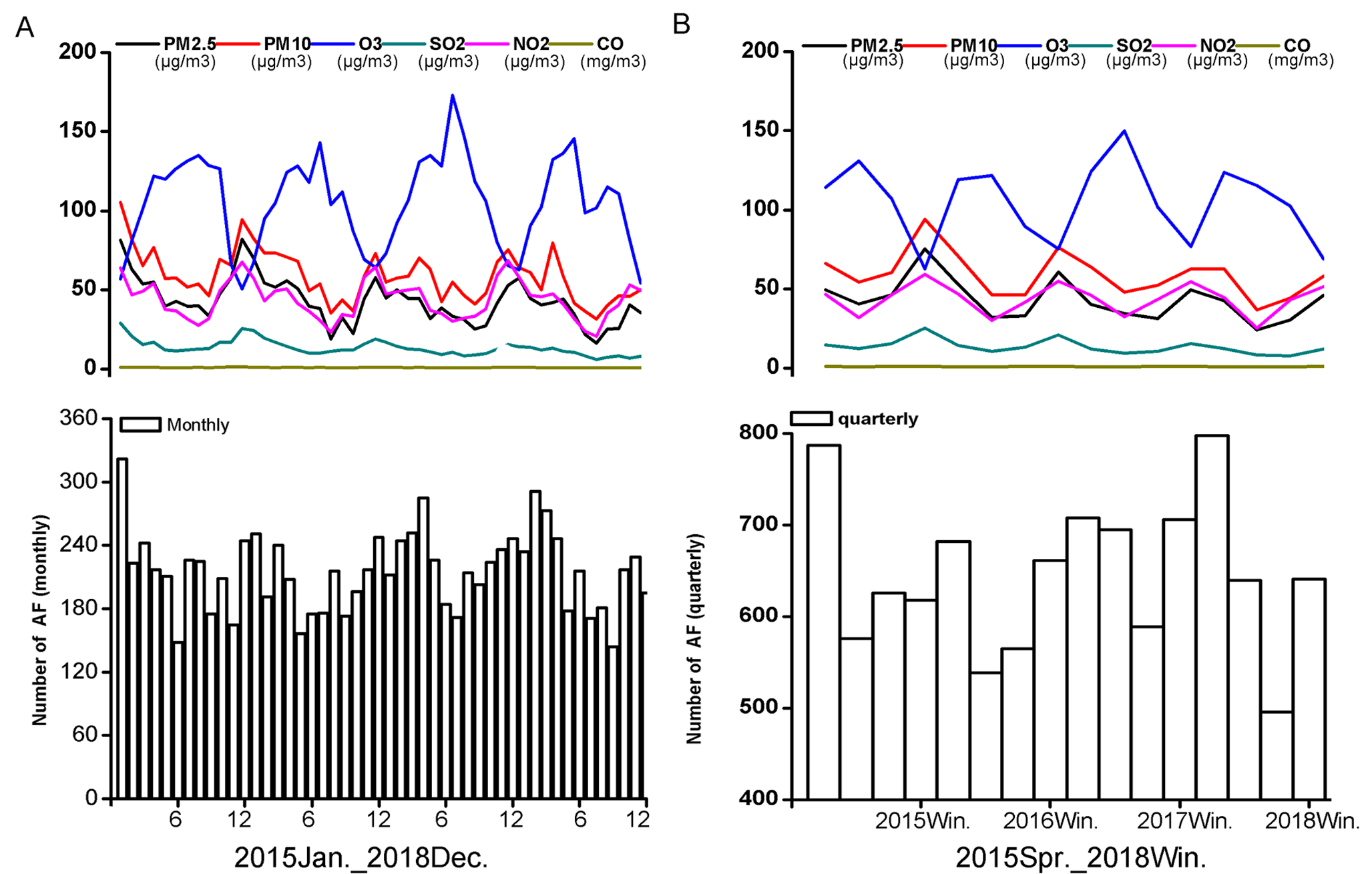

Supplement: Supplementary file 3 — Additional file 3: Fig. S1. Monthly and quarterly prevalence of AF recorded by ECG with air pollution from 2015 to 2018. We did observe a rebound of AF recorded by ECG in spring every year. And monthly incidence of AF recorded by ECG reached its peak in Januaryand February. However, the peak time in 2017 was seen in April. Besides, the monthly and quarterly concentration changes of PM2.5, PM10, O3, SO2, NO2 and CO from 2015 to 2018 were shown too. [file 40001_2023_1170_MOESM3_ESM.tif]
