# Supplementary material for: The association between air pollution and the daily hospital visits for atrial fibrillation recorded by ECG: a case-crossover study
Source: Eur J Med Res. 2023 Jun 29;28:201. doi: 10.1186/s40001-023-01170-y (PMC10308751; doi:10.1186/s40001-023-01170-y)
Supplement: Supplementary file 4 — Additional file 4: Table S3. The relationship of air pollution and meteorological measurements. [file 40001_2023_1170_MOESM4_ESM.doc]

**Supplementary table3. The relationship of air pollution and meteorological measurements.**

|  | PM2.5 | PM10 | O3 | SO2 | NO2 | CO | Average pressure | Average temperature | Average humidity | Average wind speed |
| --- | --- | --- | --- | --- | --- | --- | --- | --- | --- | --- |
| PM2.5 | 1.000 | 0.294** | 0.137** | 0.684** | 0.680** | 0.819** | 0.172** | -0.260** | -0.175** | -0.273** |
| PM10 | 0.294** | 1.000 | 0.046 | 0.021 | 0.264** | 0.096** | 0.115** | -0.096** | -0.201** | -0.141** |
| O3 | 0.137** | 0.046 | 1.000 | -0.006 | -0.201** | -0.101** | -0.432** | 0.505** | -0.315** | 0.074** |
| SO2 | 0.684** | 0.021 | -0.006 | 1.000 | 0.607** | 0.649** | 0.400** | -0.423** | -0.437** | -0.168** |
| NO2 | 0.680** | 0.264** | -0.201** | -0.607** | 1.000 | 0.703** | 0.408** | -0.462** | -0.131** | -0.524** |
| CO | 0.819** | 0.096** | -0.101** | 0.649** | 0.703** | 1.000 | 0.209** | -0.292** | -0.053* | -0.295** |
| Average pressure | 0.172** | 0.115** | -0.432** | 0.400** | 0.408** | 0.209** | 1.000 | -0.885** | -0.343** | -0.134** |
| Average temperature | -0.260** | -0.096** | 0.505** | -0.423** | -0.462** | -0.292** | -0.885** | 1.000 | 0.189** | 0.083** |
| Average humidity | -0.175** | -0.201** | -0.315** | -0.437** | -0.131** | -0.053** | -0.343** | 0.189** | 1.000 | -0.045 |
| Average wind speed | -0.273** | -0.141** | 0.074** | -0.168** | -0.524** | -0.295** | -0.134** | 0.083** | -0.045 | 1.000 |

** p<0.01, *p<0.05.
